# Supplementary material for: Squirrel: Reconstructing Semi-directed Phylogenetic Level-1 Networks from Four-Leaved Networks or Sequence Alignments
Source: Mol Biol Evol. 2025 Mar 27;42(4):msaf067. doi: 10.1093/molbev/msaf067 (PMC11979102; doi:10.1093/molbev/msaf067)
Supplement: msaf067_Supplementary_Data [file msaf067_supplementary_data.zip › Squirrel_supplementary_material.pdf]

Supplementary Material

SQUIRREL: Reconstructing semi-directed phylogenetic level-1 networks from four-leaved networks or sequence alignments

Niels Holtgrefe[1], Katharina T. Huber[2], Leo van Iersel[1], Mark Jones[1], Samuel Martin[3], and Vincent Moulton[2]

[1]*Delft Institute of Applied Mathematics, Delft University of Technology, Mekelweg 4, 2628CD, Delft, The Netherlands*
[2]*School of Computing Sciences, University of East Anglia, NR4 7TJ, Norwich, United Kingdom*
[3]*European Bioinformatics Institute, CB10 1SD, Hinxton, United Kingdom*

A Proof of consistency

In this section we prove that SQUIRREL is combinatorially consistent given an unweighted dense set of tf-quarnets:

Theorem 1. *Let $\mathcal{N}$ be a triangle-free semi-directed level-1 network and let $\mathcal{Q}$ be the set of unweighted tf-quarnets induced by $\mathcal{N}$, then* SQUIRREL *applied to $\mathcal{Q}$ reconstructs $\mathcal{N}$.*

We start by giving a formal definition (taken from Berry and Gascuel (2000), originally by Bandelt and Dress (1986)) of the tree $\mathcal{T}^*$ constructed in Section 4.3, after which we prove that it is a combinatorially consistent estimator of the blobtree of the network $\mathcal{N}$.

Given a non-trivial split $A|B$ of $\mathcal{X}$, we let $\mathcal{Q}'_{A|B}$ be the set of all possible quartets that *agree* with the split $A|B$. That is, for every $a_1, a_2 \in A$ and $b_1, b_2 \in B$, $\mathcal{Q}'_{A|B}$ contains the quartet q with split $a_1a_2|b_1b_2$. Given a (possibly non-dense) set of quartets $\mathcal{Q}'$ on $\mathcal{X}$, we let S^* be the maximal set of splits of $\mathcal{X}$ such that $\mathcal{Q}'_{A|B} \subseteq \mathcal{Q}'$ for all splits $A|B$ in S^*. In other words, S^* is the maximal set of splits such that $\mathcal{Q}'$ contains all quartets that agree with these splits. Lastly, we define the set $\mathcal{Q}^*$ as the subset of $\mathcal{Q}'$ that contains exactly these quartets, i.e. $\mathcal{Q}^* = \bigcup_{A|B \in S^*} \mathcal{Q}'_{A|B}$. In Berry and Gascuel (2000) it is shown that the set $\mathcal{Q}^*$ can also be characterized as the unique maximum subset of $\mathcal{Q}'$ that is *tree-like*, meaning that there exists a phylogenetic tree $\mathcal{T}$ on $\mathcal{X}$ with $\mathcal{Q}^*$ as the set of resolved quartets (i.e. the quartets contain a non-trivial split) it induces. This is the tree $\mathcal{T}^*(\mathcal{Q}')$ (or simply $\mathcal{T}^*$ if the set $\mathcal{Q}'$ is clear). Note that this tree is unique since a phylogenetic tree is uniquely determined by its quartets (Colonius and Schulze 1981), and thus by its resolved quartets.

We are now ready to prove that the tree $\mathcal{T}^*$ constructed by SQUIRREL is exactly the blobtree of $\mathcal{N}$, provided the tf-quarnets induced by $\mathcal{N}$ are used as input. Recall that to construct the tree $\mathcal{T}^*$ given a dense set $\mathcal{Q}$ of tf-quarnets, SQUIRREL first creates a set of quartets $\mathcal{Q}' \subseteq \mathcal{Q}$ by throwing out all the 4-cycles. This set can then be used by to obtain the tree $\mathcal{T}^*$ as described in Berry and Gascuel (2000).

Lemma A.2. *Let $\mathcal{N}$ be a triangle-free semi-directed level-1 network, let $\mathcal{Q}$ be the set of tf-quarnets induced by $\mathcal{N}$ and let $\mathcal{Q}' \subseteq \mathcal{Q}$ be the set of quartet trees in $\mathcal{Q}$. Then, the blobtree of $\mathcal{N}$ is equal to the tree $\mathcal{T}^*(\mathcal{Q}')$.*

Proof. Let $\tilde{\mathcal{Q}}$ be the set of resolved quartets (i.e. quartets with a non-trivial split) induced by the blobtree $\mathcal{T}$ of $\mathcal{N}$. It is enough to show that $\tilde{\mathcal{Q}} = \mathcal{Q}^*$ since phylogenetic trees are uniquely determined by their induced resolved quartets (Colonius and Schulze 1981) and, by definition, $\mathcal{Q}^*$ is the set of induced resolved quartets of $\mathcal{T}^*(\mathcal{Q}')$.

Let $\tilde{q} \in \tilde{\mathcal{Q}}$ be a resolved quartet with split $\tilde{a}_1\tilde{a}_2|\tilde{b}_1\tilde{b}_2$. This means that there is some non-trivial split $A|B$ in $\mathcal{T}$ (and thus in $\mathcal{N}$) with $\tilde{a}_1, \tilde{a}_2 \in A$ and $\tilde{b}_1, \tilde{b}_2 \in B$. Now let $a_1, a_2 \in A$ and $b_1, b_2 \in B$ be arbitrary. By Theorem 5.1 in Huber et al. (2024) the quarnet q with $\mathcal{L}(q) = \{a_1, a_2, b_1, b_2\}$ that is induced by $\mathcal{N}$ then has split $a_1a_2|b_1b_2$. This directly implies that there is a quartet in $\mathcal{Q}'$ with split $a_1a_2|b_1b_2$. Consequently, $\mathcal{Q}'_{A|B} \subseteq \mathcal{Q}'$ and thus $\mathcal{Q}'_{A|B} \subseteq \mathcal{Q}^*$. Because $\tilde{q}$ agrees with $A|B$ we have $\tilde{q} \in \mathcal{Q}'_{A|B}$, so we obtain that $\tilde{q} \in \mathcal{Q}^*$. Since $\tilde{q} \in \tilde{\mathcal{Q}}$ was arbitrary, $\tilde{\mathcal{Q}} \subseteq \mathcal{Q}^*$.

Now let $q^* \in \mathcal{Q}^*$ be an arbitrary resolved quartet with split $a_1^*a_2^*|b_1^*b_2^*$. By definition, q^* must be in $\mathcal{Q}'_{A|B} \subseteq \mathcal{Q}$ for some non-trivial split $A|B$ (with $a_1^*, a_2^* \in A$ and $b_1^*, b_2^* \in B$). Since $\mathcal{Q}'_{A|B} \subseteq \mathcal{Q}'$, for any $a_1, a_2 \in A$ and $b_1, b_2 \in B$ the quartet q with split $a_1a_2|b_1b_2$ is in $\mathcal{Q}'$. By (Huber et al. 2024, Thm. 5.1), this means that $A|B$ is a split in $\mathcal{N}$ and thus in $\mathcal{T}$. But q^* is a resolved quartet of $\mathcal{T}$, so $q^* \in \tilde{\mathcal{Q}}$. Since q^* was arbitrary, this shows that $\mathcal{Q}^* \subseteq \tilde{\mathcal{Q}}$. □

We continue with proving that the approach in Step B2 of SQUIRREL correctly determines the ordering of each cycle. For the rest of this section, we consider $\mathcal{Q}$ to be a dense set of unweighted tf-quarnets induced by a *sunlet network*: a semi-directed level-1 network consisting of a single cycle with pendent leaves. This will then be enough to prove the consistency at the end of this section.

Clearly, a sunlet network induces a circular ordering of its leaf set $\mathcal{Y}$. We now create an explicit formula for the distance function $D_{\mathcal{Q}}$ defined in Section 4.4, assuming the set $\mathcal{Q}$ contains tf-quarnets induced by a sunlet network. Note that a distance function D on a finite set of elements $\mathcal{Y}$ is a *metric* if (i) it is symmetric; (ii) the triangle inequality holds; (iii) the distance between two elements is zero if and only if the elements are equal.

Lemma A.3. *Let $\mathcal{N}$ be a sunlet network on $\mathcal{Y} = \{y_1, \dots, y_n\}$, let $\theta = (y_1, \dots, y_n)$ be a circular ordering of $\mathcal{Y}$ induced by $\mathcal{N}$ such that y_1 is the leaf below the reticulation. If $\mathcal{Q}$ is the set of unweighted tf-quarnets induced by $\mathcal{N}$, then $D_{\mathcal{Q}}$ is a metric on $\mathcal{Y}$ and it can be expressed as*

$$D_{\mathcal{Q}}(y_i, y_j) = \frac{1}{2} \cdot \begin{cases} 0 & \text{if } i = j; \\ n^2 - 9n - 2j^2 + (2n+4) \cdot j + 6 & \text{if } i = 1 \text{ and } i \neq j; \\ n^2 - 11n - i^2 - j^2 + (2n+1) \cdot j + 3i + 10 & \text{if } i \geq 2 \text{ and } i \neq j. \end{cases}$$

Proof. Clearly, $D_{\mathcal{Q}}$ is symmetric and two leaves have distance zero if and only if they are the same. To see that the triangle inequality holds, note that $D_{\mathcal{Q}}$ is defined as a sum of τ-values. Since those values all adhere to the triangle inequality (assuming the tf-quarnets all have weight 1), it readily follows that $D_{\mathcal{Q}}$ also has the same property. Hence, it is a metric.

We will now find the expression for the distances defined by $D_{\mathcal{Q}}$. Given two leaves y_i and y_j with $1 \leq i < j \leq n$, let $r_i = i - 2$ be the number of leaves between y_1 and y_i, and let $t_j = n - j$ be the number of leaves between y_j and y_1 (on the side of the cycle containing leaf y_n). Lastly, let $s_{ij} = j - i - 1$ be the number of leaves between y_i and y_j. See Figure A.1 for an illustration.

Now let $i = 1$ and let $i < j \leq n$ be arbitrary. Any tf-quarnet in $\mathcal{Q}$ containing both y_i and y_j will be a 4-cycle. In particular, in $\binom{s_{1j}}{2} + \binom{t_j}{2}$ of these 4-cycles, y_i and y_j will be opposite leaves. On the other hand, in $s_{1j} \cdot t_j$ of these 4-cycles they will be neighbours.

We now let $1 < i < j \leq n$ be arbitrary. In this case, any tf-quarnet in $\mathcal{Q}$ containing both y_i and y_j will either be a quartet tree or a 4-cycle. All 4-cycle tf-quarnets must contain leaf y_1 as well. Then, in $r_i + t_j$ of them y_i and y_j are opposite leaves, while in s_{ij} of them they are neighbours. All quartet trees that include y_i and y_j, do not include leaf y_1. In $\binom{r_i}{2} + \binom{t_j}{2}$ of the quartet trees, y_i and y_j are on the same side of the split, i.e. there is no '$y_i|y_j$ split'. The number of quartet trees that do have a '$y_i|y_j$ split' is $\binom{s_{ij}}{2} + r_i \cdot t_j + r_i \cdot s_{ij} + s_{ij} \cdot t_j$. Filling in the distances defined by $D_{\mathcal{Q}}$, we thus obtain for each $1 \leq i < j \leq n$:

$$D_{\mathcal{Q}}(y_i, y_j) = \begin{cases} \overbrace{\left[\binom{s_{1j}}{2} + \binom{t_j}{2}\right]}^{\#4C:y_i,y_j \text{ neighbours}} + 2 \cdot \overbrace{[s_{1j} \cdot t_j]}^{\substack{\#4C:y_i,y_j \\ \text{opposite}}} & \text{if } i = 1; \\ \underbrace{[r_i + t_j]}_{\#4C:y_i,y_j \text{ neighbours}} + 2 \cdot \underbrace{[s_{ij}]}_{\substack{\#4C:y_i,y_j \\ \text{opposite}}} + \underbrace{\left[\binom{r_i}{2} + \binom{t_j}{2}\right]}_{\#QT \text{ without } y_i|y_j \text{ split}} + 2 \cdot \underbrace{\left[\binom{s_{ij}}{2} + r_i \cdot t_j + r_i \cdot s_{ij} + s_{ij} \cdot t_j\right]}_{\#QT \text{ with } y_i|y_j \text{ split}} & \text{if } i \geq 2. \end{cases}$$

If we fill in the formulas for r_i, t_j and s_{ij}, we obtain

$$D_{\mathcal{Q}}(y_i, y_j) = \begin{cases} \left[\binom{j-2}{2} + \binom{n-j}{2}\right] + 2 \cdot [(j-2) \cdot (n-j)] & \text{if } i = 1; \\ [(i-2) + (n-j)] + 2 \cdot (j-i-1) + \left[\binom{i-2}{2} + \binom{n-j}{2}\right] & \\ \quad + 2 \cdot \left[\binom{j-i-1}{2} + (i-2) \cdot (n-j) + (i-2) \cdot (j-i-1) + (j-i-1) \cdot (n-j)\right] & \text{if } i \geq 2, \end{cases}$$

which reduces to

$$D_{\mathcal{Q}}(y_i, y_j) = \begin{cases} \binom{n-j}{2} + \binom{j-2}{2} + 2 \cdot (n-j) \cdot (j-2) & \text{if } i = 1; \\ \binom{n-j+1}{2} + \binom{i-1}{2} + 2 \cdot \binom{j-i}{2} + 2 \cdot (n-j) \cdot (j-3) + 2 \cdot (j-i-1) \cdot (i-2) & \text{if } i \geq 2. \end{cases}$$

After writing out the binomial coefficients and expanding all brackets, one obtains the desired formula for those y_i and y_j with $1 \leq i < j \leq n$. The other cases follow since $D_{\mathcal{Q}}$ is a metric. □

The explicit formula derived in the previous lemma allows us to prove that $D_{\mathcal{Q}}$ is *Kalmanson* (Kalmanson 1975) with respect to the circular ordering θ of the leaves. Such a metric D defined on a set of elements $\mathcal{Y}$ has

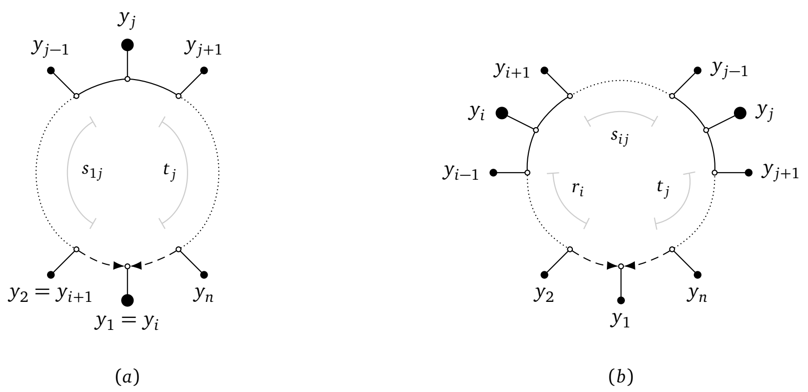

Figure A.1: Illustration of the definitions of r_i, s_{ij} and t_j in the proof of Lemma A.3. Subfigure (a) is the case where $i = 1$ and subfigure (b) is the case where $i > 1$.

the following nice property: it is easy to find an optimal TSP-tour in the complete graph on $\mathcal{Y}$ with distances defined by D. Moreover, the corresponding ordering θ defines an optimal TSP-tour. Formally, these metrics are defined as follows.

Definition A.4 (Kalmanson metric). Let $\theta = (y_1, \ldots, y_n)$ be an ordering of a finite set of elements $\mathcal{Y} = \{y_1, \ldots, y_n\}$. A metric D on $\mathcal{Y}$ is *Kalmanson* with respect to θ if both of the following conditions hold:

$$D(y_i, y_j) + D(y_k, y_l) \leq D(y_i, y_k) + D(y_j, y_l) \text{ for all } 1 \leq i < j < k < l \leq n,$$

$$D(y_i, y_l) + D(y_j, y_k) \leq D(y_i, y_k) + D(y_j, y_l) \text{ for all } 1 \leq i < j < k < l \leq n.$$

In the next lemma we prove that D_Q is a Kalmanson metric with respect to a circular ordering of the leaves induced by a sunlet network. From this it follows that TSP can be used to find such an ordering.

Lemma A.5. *Let $\mathcal{N}$ be a sunlet network on $\mathcal{Y} = \{y_1, \ldots, y_n\}$ and let $\mathcal{Q}$ be the set of unweighted tf-quarnets induced by $\mathcal{N}$. Then, a circular ordering θ of $\mathcal{Y}$ is induced by $\mathcal{N}$ if and only if θ is an optimal TSP-tour defined by $D_\mathcal{Q}$.*

Proof. Let $\theta = (y_1, \ldots, y_n)$ be an ordering of $\mathcal{Y}$ induced by $\mathcal{N}$. We will first show that $D_\mathcal{Q}$ is Kalmanson with respect to θ. Then, it will follow from Kalmanson (1975) that θ is an optimal TSP-tour. Without loss of generality, we can assume that y_1 is the leaf below the reticulation since being Kalmanson is invariant under cyclic permutations (see e.g. Deĭneko et al. 1997). We will now prove that $D_\mathcal{Q}$ is Kalmanson with respect to θ by checking the Kalmanson conditions from Definition A.4. For easier notation, we multiply the expressions by 2. Now let $1 \leq i < j < k < l \leq n$ be arbitrary. Using the explicit formula of Lemma A.3, we then distinguish between the cases where $i = 1$ and $i > 1$. Note that the $n^2 - 9n + 6$ and $n^2 - 11n + 10$ parts cancel out in all conditions.

Case 1: $i = 1$. To prove the first condition we use that $j < k$:

$$\begin{aligned} 2 \cdot \big(D_\mathcal{Q}(y_i, y_k) + D_\mathcal{Q}(y_j, y_l) - D_\mathcal{Q}(y_i, y_j) - D_\mathcal{Q}(y_k, y_l)\big) &= \big[-2k^2 + (2n+4) \cdot k\big] + \big[-j^2 - l^2 + (2n+1) \cdot l + 3j\big] \\ &\quad - \big[-2j^2 + (2n+4) \cdot j\big] - \big[-k^2 - l^2 + (2n+1) \cdot l + 3k\big] \\ &= (k - j) \cdot (2n + k - j + 1) > 0. \end{aligned}$$

For the second condition, we use the fact that $3 < k < l$:

$$\begin{aligned} 2 \cdot \big(D_\mathcal{Q}(y_i, y_k) + D_\mathcal{Q}(y_j, y_l) - D_\mathcal{Q}(y_i, y_l) - D_\mathcal{Q}(y_j, y_k)\big) &= \big[-2k^2 + (2n+4) \cdot k\big] + \big[-j^2 - l^2 + (2n+1) \cdot l + 3j\big] \\ &\quad - \big[-2l^2 + (2n+4) \cdot l\big] - \big[-j^2 - k^2 + (2n+1) \cdot k + 3j\big] \\ &= (l - k) \cdot (l + k - 3) > 0. \end{aligned}$$

Case 2: $i > 1$. The first condition follows from the fact that $k > j$ and $2n > 2$:

$$\begin{aligned} 2 \cdot \big(D_\mathcal{Q}(y_i, y_k) + D_\mathcal{Q}(y_j, y_l) - D_\mathcal{Q}(y_i, y_j) - D_\mathcal{Q}(y_k, y_l)\big) &= \big[-i^2 - k^2 + (2n+1) \cdot k + 3i\big] + \big[-j^2 - l^2 + (2n+1) \cdot l + 3j\big] \\ &\quad - \big[-i^2 - j^2 + (2n+1) \cdot j + 3i\big] - \big[-k^2 - l^2 + (2n+1) \cdot l + 3k\big] \\ &= (2n - 2) \cdot (k - j) > 0. \end{aligned}$$

The second condition is trivial:

$$\begin{aligned} 2 \cdot \big(D_\mathcal{Q}(y_i, y_k) + D_\mathcal{Q}(y_j, y_l) - D_\mathcal{Q}(y_i, y_l) - D_\mathcal{Q}(y_j, y_k)\big) &= \big[-i^2 - k^2 + (2n+1) \cdot k + 3i\big] + \big[-j^2 - l^2 + (2n+1) \cdot l + 3j\big] \\ &\quad - \big[-i^2 - l^2 + (2n+1) \cdot l + 3i\big] - \big[-j^2 - k^2 + (2n+1) \cdot k + 3j\big] \\ &= 0. \end{aligned}$$

It remains to show that any circular ordering ϕ of $\mathcal{Y}$ that is not induced by $\mathcal{N}$ is not an optimal TSP-tour. To see this, we argue that the total TSP-distance of any such ordering ϕ can always be decreased by swapping two specific adjacent leaves. In particular, any such ordering ϕ will have four leaves $\{y_i, y_j, y_k, y_l\}$ (with $1 \leq i < j < k < l \leq n$) adjacent in the ordering ϕ but ordered as (y_i, y_k, y_j, y_l) (if $i \neq 1$), or ordered as (y_1, y_k, y_j, y_l) or (y_j, y_1, y_k, y_l) (if $i = 1$). In all three cases, we can swap two of these adjacent leaves to decrease the total distance, since the corresponding three Kalmanson inequalities are strict inequalities. □

We are now ready to present the proof of Theorem 1.

Proof of Theorem 1. Since the set of tf-quarnets $\mathcal{Q}$ is induced by the network $\mathcal{N}$, we know by Lemma A.2 that the tree $\mathcal{T}^*$ (as constructed in Step A1) is equal to the blobtree of $\mathcal{N}$. The tree $\mathcal{T}_1$ (in Step A2) is a *refinement* of $\mathcal{T}^*$. That is, $\mathcal{T}^*$ can be obtained from $\mathcal{T}_1$ by contracting edges. Recall that the tree $\mathcal{T}^*$ is the unique most refined tree on $\mathcal{X}$ such that no tf-quarnet in $\mathcal{Q}$ contradicts a split of $\mathcal{T}^*$. This means that if we keep contracting the least supported split of $\mathcal{T}_1$ (see equation (4) in Step A3 of Section 4.3), we eventually end up with $\mathcal{T}^*$. Therefore, the tree $\mathcal{T}^*$ (and thus the blobtree of $\mathcal{N}$) is part of the sequence $(\mathcal{T}_1, \dots, \mathcal{T}_{n-3})$.

Next, we show that given the blobtree $\mathcal{T}^*$ of $\mathcal{N}$ (and assuming the set $\mathcal{Q}$ is induced by $\mathcal{N}$), Step B of Squirrel correctly constructs the network $\mathcal{N}$. We first show this is true when $\mathcal{N}$ is a sunlet network (and so $\mathcal{T}^*$ is an unresolved tree with a single internal vertex). Then, the set $\tilde{\mathcal{Q}}_v$ constructed in Step B1 is the same as the original set $\mathcal{Q}$ of tf-quarnets (up to the relabeling defined by f). From Lemma A.5 we then know that the optimal TSP-tour using the distances $D_{\tilde{\mathcal{Q}}_v}$ will correspond to the circular ordering induced by $\mathcal{N}$. Whenever the sunlet network $\mathcal{N}$ has at least five leaves, only the leaf below the reticulation will appear in every 4-cycle tf-quarnet induced by $\mathcal{N}$. On the other hand, if $\mathcal{N}$ has only four leaves it only induces one tf-quarnet: a 4-cycle with the correct leaf below its reticulation. Hence, Squirrel correctly picks the reticulation vertex in Step B3. Thus, in the case that $\mathcal{N}$ is a sunlet network Squirrel constructs $\mathcal{N}$ from $\mathcal{T}^*$.

Whenever $\mathcal{N}$ is not a sunlet network, the proof is similar. To be precise, for every internal vertex v with induced partition $Y_1|\dots|Y_s$, let $\tilde{y}_i$ be an arbitrary leaf of Y_i. Then, up to the relabeling defined by f, the set $\tilde{\mathcal{Q}}_v$ (as constructed in Step B1) is equal to the set of tf-quarnets induced by the sunlet network $\mathcal{N}|_{\{\tilde{y}_1,\dots,\tilde{y}_s\}}$ (the restriction of $\mathcal{N}$ to $\{\tilde{y}_1, \dots, \tilde{y}_s\}$). Using a similar argument as above, Step B3 then correctly reconstructs the cycle in the sunlet network $\mathcal{N}|_{\{\tilde{y}_1,\dots,\tilde{y}_s\}}$. From this, it follows that we replace v in $\mathcal{T}^*$ by the correct cycle.

Since the blobtree $\mathcal{T}^*$ of $\mathcal{N}$ was part of the sequence $(\mathcal{T}_1, \dots, \mathcal{T}_{n-3})$, exactly one of the candidate networks $(\mathcal{N}_1, \dots, \mathcal{N}_{n-3})$ will be our original network $\mathcal{N}$. By Frohn et al. (2025) we know that tf-quarnets are enough to encode a triangle-free semi-directed level-1 network. Hence, this will be the only network with a (weighted) tf-quarnet consistency score of 1 and so Squirrel returns the correct network $\mathcal{N}$. □

Whereas in Theorem 1 we showed that Squirrel reconstructs a network from its set of tf-quarnets, in practice, the input will not be a set of tf-quarnets coming from one unique network. In the following lemma we prove that even if the input tf-quarnets do not come from one unique network, Squirrel is still guaranteed to return a triangle-free semi-directed level-1 network.

Lemma A.6. *Let $\mathcal{Q}$ be a dense set of weighted tf-quarnets on $\mathcal{X}$, then* Squirrel *applied to $\mathcal{Q}$ returns a triangle-free semi-directed level-1 network on $\mathcal{X}$.*

Proof. Step A (Algorithm 1) of Squirrel relies on algorithms from Berry and Gascuel (2000) and Grünewald et al. (2009). It follows from those papers that Algorithm 1 always returns a sequence of phylogenetic trees on $\mathcal{X}$. To prove the lemma, we now need to show that Step B of Squirrel (Algorithm 2) returns a triangle-free semi-directed level-1 network on $\mathcal{X}$ for any phylogenetic tree $\mathcal{T}$ on $\mathcal{X}$. Since Algorithm 2 only alters the tree $\mathcal{T}$ by replacing internal vertices of degree at least 4 by cycles with reticulations, the resulting network will always be level-1 and triangle-free. It remains to prove that the resulting network is a *valid* semi-directed network (i.e. a network that has a valid root-location, or equivalently, a network with no two reticulations oriented towards each other or towards the optional outgroup).

Clearly, when the first high-degree node is replaced by a cycle in the first iteration of the algorithm, there always is a location for the reticulation vertex that results in a partial network with a valid root-location. We can thus inductively assume that at the start of the other iterations, when we want to replace some internal vertex v by a cycle, the partial network constructed in the previous iteration has a valid root-location at some edge (or in case of a specified outgroup, the specific edge incident to the outgroup). Hence, the partial network can be rooted at this edge, forming a non-binary rooted phylogenetic network. We can then expand the high-degree node in this directed acyclic graph that corresponds to v in such a way that the resulting rooted phylogenetic network remains a directed acyclic graph. Thus, when we replace v by a cycle in the partially constructed semi-directed network, there is a location for the reticulation such that the partial network remains valid, which proves the lemma. □

B Random network generator

In this section we provide a concise description of the algorithm that was used to generate the random semi-directed level-1 networks in Section 2.1. The algorithm takes as input a number of leaves n and a number of reticulations $r \geq 1$. In the special case that $r = 0$, we simply generate a random network with $r = 1$ reticulation and randomly turn it into a phylogenetic tree by deleting one of the reticulation edges (and suppressing the resulting degree-2 vertex). We first outline the general structure of the algorithm, while in the last paragraph we explain how the reticulation number is enforced. We emphasize that this algorithm is able to generate any semi-directed level-1 network on n leaves and with k reticulations, up to the labeling of the leaves.

The algorithm starts by building a random (possibly non-binary) phylogenetic tree on n leaves. In particular, it first generates a random tree on n vertices (by constructing a random spanning tree on a complete graph with n vertices), after which it attaches one leaf to every vertex. By suppressing all degree-2 vertices this results in a (possibly non-binary) tree with n leaves. Such a tree is transformed into a triangle-free semi-directed level-1 network by replacing every internal vertex of degree at least 4 by a random cycle. Finally, a random vertex of the cycle is assigned as a reticulation (while ensuring the resulting semi-directed network has a valid root location).

To enforce the correct reticulation number r in the resulting network, we ensure that the initial tree has exactly r internal vertices of high degree (i.e. a degree of at least 4). To this end, we iteratively adjust the tree until it satisfies this condition. In particular, if our initial tree has more than r high degree internal vertices, we contract the unique path between two random high degree internal vertices (without another high degree vertex on this path). This decreases the number of internal vertices with high degree by exactly one. On the other hand, if our initial tree has less than r of these high-degree internal vertices, we contract a random edge between two degree-3 nodes to create a new high degree vertex. This process is repeated until the tree has exactly r high-degree internal vertices.

References

Bandelt HJ, Dress A. 1986. Reconstructing the shape of a tree from observed dissimilarity data. *Advances in Applied Mathematics*. 7:309–343.

Berry V, Gascuel O. 2000. Inferring evolutionary trees with strong combinatorial evidence. *Theoretical Computer Science*. 240:271–298.

Colonius H, Schulze HH. 1981. Tree structures for proximity data. *British Journal of Mathematical and Statistical Psychology*. 34:167–180.

Deĭneko VG, van der Veen JA, Rudolf R, Woeginger GJ. 1997. Three easy special cases of the euclidean travelling salesman problem. *RAIRO-Operations Research*. 31:343–362.

Frohn M, Holtgrefe N, van Iersel L, Jones M, Kelk S. 2025. Reconstructing semi-directed level-1 networks using few quarnets. *Journal of Computer and System Sciences*. To appear.

Grünewald S, Moulton V, Spillner A. 2009. Consistency of the qnet algorithm for generating planar split networks from weighted quartets. *Discrete Applied Mathematics*. 157:2325–2334.

Huber KT, van Iersel L, Jones M, Moulton V, Veenema-Nipius L. 2024. When are quarnets sufficient to reconstruct semi-directed phylogenetic networks? arXiv:2408.12997.

Kalmanson K. 1975. Edgeconvex circuits and the traveling salesman problem. *Canadian Journal of Mathematics*. 27:1000–1010.
